# Deletion of LRP1 From Astrocytes Modifies Neuronal Network Activity in an *in vitro* Model of the Tripartite Synapse

**DOI:** 10.3389/fncel.2020.567253

**Published:** 2021-01-14

**Authors:** Ramona Romeo, Kristin Glotzbach, Anja Scheller, Andreas Faissner

**Affiliations:** ^1^Department of Cell Morphology and Molecular Neurobiology, Ruhr-University Bochum, Bochum, Germany; ^2^Department of Molecular Physiology, Center for Integrative Physiology and Molecular Medicine (CIPMM), University of Saarland, Homburg, Germany

**Keywords:** astrocytes, astrocytic heterogeneity, synaptogenesis, *in vitro* knockout model LRP1, neuronal plasticity, tripartite synapse

## Abstract

The low-density lipoprotein receptor-related protein 1 (LRP1) is a transmembrane receptor that binds over 40 potential ligands and is involved in processes such as cell differentiation, proliferation, and survival. LRP1 is ubiquitously expressed in the organism and enriched among others in blood vessels, liver, and the central nervous system (CNS). There, it is strongly expressed by neurons, microglia, immature oligodendrocytes, and astrocytes. The constitutive LRP1 knockout leads to embryonic lethality. Therefore, previous studies focused on conditional LRP1-knockout strategies and revealed that the deletion of LRP1 causes an increased differentiation of neural stem and precursor cells into astrocytes. Furthermore, astrocytic LRP1 is necessary for the degradation of Aβ and the reduced accumulation of amyloid plaques in Alzheimer’s disease. Although the role of LRP1 in neurons has intensely been investigated, the function of LRP1 with regard to the differentiation and maturation of astrocytes and their functionality is still unknown. To address this question, we generated an inducible conditional transgenic mouse model, where LRP1 is specifically deleted from GLAST-positive astrocyte precursor cells. The recombination with resulting knockout events was visualized by the simultaneous expression of the fluorescent reporter tdTomato. We observed a significantly increased number of GLT-1 expressing astrocytes in LRP1-depleted astrocytic cultures in comparison to control astrocytes. Furthermore, we investigated the influence of astrocytic LRP1 on neuronal activity and synaptogenesis using the co-culture of hippocampal neurons with control or LRP1-depleted astrocytes. These analyses revealed that the LRP1-deficient astrocytes caused a decreased number of single action potentials as well as a negatively influenced neuronal network activity. Moreover, the proportion of pre- and postsynaptic structures was significantly altered in neurons co-cultured with LPR1-depleted astrocytes. However, the number of structural synapses was not affected. Additionally, the supernatant of hippocampal neurons co-cultured with LRP1-deficient astrocytes showed an altered set of cytokines in comparison to the control condition, which potentially contributed to the altered neuronal transmission and synaptogenesis. Our results suggest astrocytic LRP1 as a modulator of synaptic transmission and synaptogenesis by altering the expression of the glutamate transporter on the cell surface on astrocytes and the release of cytokines *in vitro*.

## Introduction

The low-density lipoprotein receptor-related protein 1 (LRP1) is a transmembrane receptor and part of the low-density lipoprotein receptor (LDL) family (May and Herz, 2003; Boucher and Herz, 2011). LRP1 consists of two covalently bound subunits, an 85 kDa intracellular β-chain and the 515 kDa extracellular α-chain with four ligand-binding sites. The physiological relevance of LRP1 becomes apparent through the more than 40 potential ligands (Boucher and Herz, 2011; Bres and Faissner, 2019), including Apolipoprotein E (ApoE), α2-macroglobulin, tissue plasminogen activator (tPa), and amyloid precursor protein (APP), just to name a few (Orth et al., 1992; Lillis et al., 2008). Binding of the ligand to LRP1 leads to endocytosis of the whole receptor–ligand–complex (Willnow et al., 2012). Therefore, LRP1 is involved in several cellular processes such as cell proliferation, differentiation and lipid metabolism. In general, LRP1 is ubiquitously expressed, but strongly enriched in the liver, lung, blood vessels, and in the central nervous system (CNS). There, it is expressed by neurons, microglia, immature oligodendrocytes, and astrocytes (May et al., 2004; Liu et al., 2010; Boucher and Herz, 2011; Auderset et al., 2016; Schäfer et al., 2019). LRP1 is critical for development, proved by the constitutive knockout causing embryonic lethality (Herz et al., 1993). Therefore, several targeted LRP1-knockout models have been developed. The conditional knockout of LRP1 in cortical and spinal cord-derived neural stem and precursor cells (NSPCs) *in vitro* produced an increased number of glial fibrillary acidic protein (GFAP)-positive astrocytes, as well as a decreased number of oligodendrocytes and neurons (Safina et al., 2016). Furthermore, the deletion of LRP1 in neurons caused hyperactivity, dystonia, and increased neurodegeneration in the cortex and hippocampus of mice (May et al., 2004; Liu et al., 2010). The functions of LRP1 in regard to differentiation and functionality of astrocytes have, however, not been intensively investigated so far. It is known that astrocytic LRP1 plays a critical role in brain Aβ clearance involved in Alzheimer’s disease (AD) pathogenesis (Liu et al., 2017). The knockdown of LRP1 in primary astrocytes resulted in decreased cellular Aβ uptake and degradation. Furthermore, the silencing of LRP1 was accompanied by the downregulation of several Aβ-degrading enzymes, like matrix metalloproteases MMP2 and MMP9. The impaired Aβ clearance caused by the astrocytic LRP1 knockdown led to an accelerated amyloid plaque deposition (Liu et al., 2017). The authors concluded that the expression of LRP1 and its function in astrocytes could be an effective strategy to counter the amyloid plaque accumulation in AD. Furthermore, Bres et al. (2020) investigated the deletion of *Lrp1* from embryonic cortical radial glia stem cells located in the telencephalon and observed a severe epileptic phenotype accompanied by an altered differentiation of astrocytic subpopulations. This resulting epileptic phenotype had not been described in knockout models where *Lrp1* had been deleted from other cell types, such as neurons or oligodendrocytes. Therefore, we aimed to investigate whether the deletion of LRP1 in the postnatal astrocytic lineage might alter neuronal transmission, eventually leading to hyperexcitability and epilepsy. The physiological functions of LRP1 in the astrocyte lineage had not been addressed so far.

Therefore, we generated a new mouse model, where LRP1 was specifically deleted from glutamate aspartate transporter (GLAST)-positive astrocyte precursor cells. We mated the floxed reporter mouse line (Mori et al., 2006; Madisen et al., 2010) with the LRP1^fl/fl^ mice (Rohlmann et al., 1996) to obtain an inducible conditional transgenic mouse model. The reporter tdTomato allowed tracing of recombination and therefore knockout events. We analyzed the maturation of astrocytes *in vitro* via the expression of stage-specific astrocytic markers. To investigate the influence of astrocytic LRP1 on neuronal activity and synaptogenesis, we co-cultured hippocampal wild-type neurons with either wild-type or LRP1-depleted astrocytes. In this approach, we analyzed the number of pre- and postsynapses, as well as neuronal network activity and spontaneous action potentials using microelectrode arrays (MEAs). Our results highlight astrocytic LRP1 as a novel regulator of directed neuronal network activity and modulator of synaptogenesis *in vitro*.

## Materials and Methods

### Animals

This study was performed under the animal license of the State Agency for Nature, Environment and Consumer Protection Northrhine-Westphalia (Landesamt fuer Umweltschutz, Naturschutz und Verbraucherschutz; file number: 84-02.04.2016.A482). The animals were housed under a 12-h light–dark cycle with constant access to food and water.

LRP1^fl/fl^ (B6;129S7-LRP1^TM 2Her^/J; Jackson Laboratories; Catalog No. JAX:012604; RRID: IMSR_JAX:012604; MGI: J:76281) were obtained from Jackson Laboratory. Knockin GLAST-Cre^ERT2^ mice [Slc1a3^TM 1(Cre/ERT2)Mgoe^; MGI: 3830051] were mated with Rosa26 reporter mice [Ai14, Gt(ROSA)26Sor^TM 14(CAG–tdTomato)Hze^; MGI: 3809524] to visualize recombination events (see Supplementary Figure 1). To generate the new LRP1-depleted mouse line, the LRP1^fl/fl^ mice were crossbred with the GLAST^Cre/ERT2^Rosa26^fl/fl^ mice. The genotyping was performed as already described in Ulc et al. (2019). Briefly, genomic DNA was isolated out of tail biopsies with 200 μl of *DirectPCR*^®^ *Lysis Reagent Tail* (Peqlab, VWR Life Science, Radnor, PA, United States) and 0.2 mg/ml Proteinase-K (Sigma-Aldrich, Chemie GmbH, Munich, Germany) at 55°C and 350 rpm overnight. The digestion was stopped at 85°C for 45 min and 350 rpm. The *Lrp1* gene was amplified according to the Jackson Laboratory protocol (forward primer: 5′-C ATACCCTCTTCAAACCCCTTCCTG-3′; reverse primer: 5′-GC AAGCTCTCCTGCTCAGACCTGGA-3′). The *Glast* wild- type allele was amplified with specific GLAST primer (forward: 5′-GAGGCACTTGGCTAGGCTCTGAGGA-3′; reverse: 5′-GA GGAGATCCTGACCGATCAGTTGG-3). The expression of the Cre recombinase was detected with a Cre primer (5′-GGTGTACGGTCAGTAAATTGGACAT-3′). The floxed Rosa26 alleles were amplified with the specific primer forward (5′-CCGAAAATCTGTGGGAAGTC-3′) and reverse (5′-CTGTT CCTGTACGGCATGG-3′), whereas the wild-type alleles were amplified with the forward (5′-AAGGGAGCTG CAGTGGAGTA-3′) and reverse primer (5′-GGCATTA AAGCAGCGTATCC-3′). Animals referred to as knockout animals displayed the genotype GLAST^Cre/wt^Rosa26^fl/fl^LRP1^fl/fl^, whereas control animals had the genotype GLAST^Cre/wt^Rosa26^fl/fl^LRP1^wt/wt^.

For the co-culture of hippocampal neurons together with either LRP1-deficient or wild-type astrocytes, the newly generated LRP1-knockout line as well as C57BL/6J (Jackson Laboratories; Catalog No. JAX:000664; RRID: IMSR_JAX:000664; and MGI: 3028467) wild-type animals were compared.

### Isolation and Cultivation of Primary Murine Astrocytes

Astrocytes were obtained by the dissection of the cortex of postnatal day P0–P3 knockout or control animals following an established protocol (Gottschling et al., 2016a, 2019), with minor alterations. Cortices of one animal were cultured in a T25 flask (Sarstedt, Nuembrecht, Germany), separately according to the respective genotype. Recombination could not be induced in early postnatal pups via lactating mothers; therefore, reporter-positive astrocytes were not available for FACS sorting. For this reason, confluent genetically modified astrocytes were treated with 1 μM of 4-hydroxy-tamoxifen (4-OH-TAM; Sigma-Aldrich, Catalog No. t5648) via the application to the medium once a day for four consecutive days to induce the activation of the Cre recombinase in culture.

### Cultivation of Hippocampal Neurons

For the cultivation of hippocampal neurons, wild-type brains from E15.5 embryos were dissected and the hippocampi were prepared. The cultured neurons were cultured with either wild-type or LRP1-deficient astrocytes in an insert co-culture model as described previously (Gottschling et al., 2016a, 2019).

### Multielectrode Array (MEA)

The neuronal activity of hippocampal neurons co-cultured with astrocytes was measured 14 and 21 days *in vitro* (div). The spontaneous activity was measured for 10 min using the MC_Rack software (Version 3.9.0, Multi Channel Systems, Reutlingen, Germany). The spikes, spontaneous single action potentials, and bursts were analyzed. Therefore, we used the already established protocol by Geissler and Faissner (2012) and Hales et al. (2010).

### Differentiation Assay

Directly after the treatment with 4-OH-TAM for four consecutive days, astrocytes were plated on poly-D-lysine (PDL; 10 μg/μl, Sigma-Aldrich, Catalog No. PD899)-coated four-well dishes at a density of 5,000 cells per well. The medium was exchanged every 7 days. After 3, 5, 7, 14, and 21 days post plating (dpp), cells were fixed for 10 min with 4% (v/v) paraformaldehyde (PFA, Carl Roth, Karlsruhe Germany) and intensively washed with phosphate-buffered saline (PBS; 137 mM sodium chloride, 3 mM kalium chloride, 6.5 mM disodium hydrogen phosphate, and 1.5 mM potassium dihydrogen phosphate; pH 7.3) and used for immunocytochemical analysis. Alternatively, 4-OH-TAM-treated astrocytes were also plated at a density of 580,000 cells per cm^2^ in PDL-coated cell culture dishes for analogous time periods and lysed for either Western blot or PCR analysis.

### Immunocytochemistry of the Differentiation Assay

The astrocytes were immunocytochemically stained as previously described (Safina et al., 2016; Ulc et al., 2019). Briefly, fixed astrocytes were washed three times with PBT-1 [10% (w/v) bovine serum albumin (BSA; Sigma-Aldrich, Catalogue-No.: A7030), 0.1% (v/v) Triton-X 100 in PBS] for 10 min. Afterward, primary antibodies were diluted in PBT-1 and the fixed astrocytes were incubated for 30 min at room temperature. As primary antibodies, we used the following: LRP1 (1:500, Abcam, Catalog No. ab92544; RRID: AB_2234877), GFAP (1:300, DAKO, Catalog No. Z0334; RRID: AB_10013382), GLT-1 (EAAT1; 1:100, Santa Cruz, Catalog No. sc-365634; RRID: AB_10844832), and phospho-Histone H3 (PH3; 1:100, Millipore, Catalog No. 06-570; RRID: AB_310177). Further on, the cells were washed thrice with PBS/A [0.1% bovine serum albumin (w/v) (BSA; Sigma-Aldrich, Catalog No. A7030) in PBS] for 10 min. Species-specific secondary antibodies coupled with Cy2 (1:250; Goat anti-mouse Cy2, Jackson Immuno Research Labs, Catalog No. 115-545-044; RRID: AB_2338844; Goat anti-rabbit Cy2, Jackson Immuno Research Labs, Catalog No. 111-545-045; RRID: AB_2338049) and the nuclear marker Hoechst (Sigma-Aldrich, Catalog No. 94403) were diluted in PBS/A and added to the cells for 30 min at room temperature in the dark. Next, the cells were washed three times with PBS for 10 min and mounted with 50% (v/v) PBS and 50% (v/v) of glycerol (PBS/glycerol) for microscopy. Pictures were taken with the Axioplan 2 imaging from Zeiss (Oberkochen, Germany). For one N, six pictures were taken at a 100-fold magnification. Therefore, two peripheral areas on the left side of the well and two peripheral areas on the right side of the well were chosen. Additionally, two pictures were taken in the center of the well. The area of GFAP-positive astrocytes was analyzed with ImageJ. Hence, five GFAP- and tdTomato-positive astrocytes were randomly chosen in one well. Following from this, 30 randomly chosen astrocytes were analyzed per N.

### Immunocytochemistry of Synapses

To stain the pre- and postsynapses of hippocampal neurons, the cells were fixed with 4% (v/v) PFA for 10 min after 14 and 21 div. The staining procedure was performed as already described in Gottschling et al. (2016b). Briefly, the neurons were washed with PBS and subsequently incubated with 25 mM glycine for 20 min before the cells were blocked with blocking buffer [10% (v/v) horse serum (Gibco, Catalog No. 16050-122), 0.1% (v/v) Triton X-100 in PBS] for 1 h. Afterward, the primary antibodies (Bassoon; 1:1000; Synaptic Systems, Catalog No. 141003; RRID: AB_887697 and PSD95; 1:500; Millipore, Catalog No. MAB1598; RRID: AB_1121285) were diluted in blocking buffer and the incubation occurred for 1 h at room temperature in a wet chamber. Next on, the neurons were washed thrice with blocking buffer before the cells were incubated with the species-specific secondary antibodies (Goat anti-mouse Cy2, 1:250, Jackson Immuno Research Labs, Catalog No. 115-545-044; RRID: AB_2338844; Goat anti-rabbit Cy3, 1:500, Jackson Immuno Research Labs, Catalog No. 111-165-045; RRID: AB_2338003) for 1 h in a dark wet chamber. Afterward, the cells were washed twice with blocking buffer, once with PBS and distilled water. At last, the neurons were covered with Immu-Mount (Thermo Scientific, Waltham, MA, Catalog No. 9990402). Images were taken with Laser Scanning Microscope Axiovert 200 M by Zeiss using a 630-fold magnification.

### RNA Isolation, cDNA Synthesis, and Real-Time PCR Analysis

Cells were lysed at specific timepoints with lysis buffer (Gene Elute Mammalian Total RNA Miniprep Kit; Sigma-Aldrich, Catalog No. RTN350-1KT) for RNA isolation. The total RNA was extracted as described in the manufacturer’s manual. Next, the whole RNA was used to synthesize cDNA with the First-Strand cDNA Synthesis Kit (Fermentas, Waltham, MA, Catalog No. K1612) following the instructions. Real-time PCR analysis was performed with specific primers (see Table 1). For normalization and as a housekeeping gene, β*-actin* was used and the analysis was performed with the PCR cycler Mastercycler gradient by Eppendorf AG (Hamburg, Germany).

**TABLE 1:** Genes monitored in the expression profile analysis of LRP1-deficient astrocytes in comparison to control astrocytes and hippocampal neurons *in vitro*.

Gene	Primer sequence	GenBank nos.
β*-actin*	F: 5′-TATGCCAACACAGTGCTGTCTGGTGG-3′	NM_007393.5
	R: 5′-TAGAAGCATTTGCGGTGGACAATGG-3′	
*Aldh1l1*	F: 5′-GGAAGTTGAGAGGGGAGGAC-3′	BC030730.1
	R: 5′-GGAAGTTGAGAGGGGAGGAC-3′	
*Aquaporin-4*	F: 5′-TTGCTTTGGATCAGCATTG-3′	NM_009700.3
	R: 5′-TGAGCTCCACATCAGGACAG-3′	
*C2*	F: 5′-GCGGAGATCCCCAGGTAGTA-3′	NM_013484.2
	R: 5′-GTGGAAGTCCCTTGGCAGAA-3′	
*C3*	F: 5′-GCCCATCCCTACAGCACTAT-3′	NM_009778.3
	R: 5′-ATCCACCCACACAGAGTCAG-3′	
*Fgfr3*	F: 5′-GTCCTGTTCTGGCCAATGTT-3′	NM_001205270.1
	R: 5′-GTTTCTGGCAGCCAAGTCTC-3′	
*Gfap*	F: 5′-CGACTATCGCCGCCAACTGC-3′	NM_001131020.1
	R: 5′-GCGATCTCGATGTCCAGGGCT-3′	
*Glast*	F: 5′-GGCGGCCCTAGATAGTAAGG-3′	XM_021208184.2
	R: 5′-AGAGTCTCCATGGCCTCTGA-3′	
*Glt-1*	F: 5′-ATGATCATGTGGTACTCCCCTC-3′	NM_001077514.4
	R: 5′-TTGTCGTCGTAAATGGACTGC-3′	
*Gria1*	F: 5′-CCGTTGACACATCCAATCAG-3′	NM_001113325.2
	R: 5′-GTTGGCGAGGATGTAGTGGT-3′	
*Gria2*	F: 5′-AACGGCGTGTAATCCTTGAC-3′	NM_013540.3
	R: 5′-CTCCTGCATTTCCTCTCCTG-3′	
*Grin1*	F: 5′-CGGCTCTTGGAAGATACAGC-3′	NM_008169.3
	R: 5′-TTGTAGACGCGCATCATCTC-3′	
*Grin2a*	F: 5′-GCTGTCAGCACTGAATCCAA-3′	NM_008170.3
	R: 5′-ATCCCTGGGAGAACTTGCTT-3′	
*Grin2b*	F: 5′-GTGAGAGCTCCTTTGCCAAC-3′	NM_008171.3
	R: 5′-GGGTTGGACTGGTTCCCTAT-3′	
*Lcn2*	F: 5′-ACTTGATCCCTGCCCCATCT-3′	NM_008491.1
	R: 5′-AAGCGGGTGAAACGTTCCTT-3′	
*Lrp1*	F: 5′-GGTAGTTGTTTCCTCAATGCTC-3′	NM_008512.2
	R: 5′-TGTTGCTGACTAACAACCTGCT-3′	
*Lrp2*	F: 5′-CTTCTGATGAGTCCGCTTGC-3′	NM_001081088.2
	R: 5′-AGTTCCCATTGCTGCACTTG-3′	
*Lrp4*	F: 5′-AGAAGTGTCAGATGGTGCGA-3′	NM_172668.3
	R: 5′-TTAAGGTTGGCACGGAGGAT-3′	
*Nestin*	F: 5′-CTCGAGCAGGAAGTGGTAGG-3′	NM_016701.3
	R: 5′-GTTAGCGCTGCCRCRAGACC-3′	
*S100*	F: 5′-TGTCTTCCACCAGTACTCCG-3′	NM_009115.3
	R: 5′-ACTCCTGGAAGTCACACTCC-3′	
*Serpina3n*	F: 5′-AGAAGACTTCCCTGATGCCC-3′	NM_009252.2
	R: 5′-AGAAGACTTCCCTGATGCCC-3′	

*The used primers were self-designed with the according nucleotide sequence (see GenBank nos.).*

### Western Blotting

The astrocytes were lysed at specific timepoints with radioimmunoprecipitation assay buffer [RIPA; 10 mM Tris–HCl (pH 8.0), 1 mM ethylenediaminetetraacetic acid (EDTA), 0.5 mM ethylene glycol-bis(β-aminoethyl ether)-N,N,N′,N′-tetraacetic acid (EGTA), 1% (v/v) Triton X-100, 0.1% (w/v) sodium deoxycholate, 0.1% (v/v) sodium dodecyl sulfate (SDS), and 140 mM NaCl, Sigma-Aldrich] with 1% (v/v) of the protease inhibitor phenylmethylsulfonyl fluoride (PMSF) and 1% (v/v) aprotinin (APR). Cell scrapers were used to detach the cells from the cell culture dishes. The cell lysates were stored at −20°C until further use. The Western blot analysis was performed as already described in Hennen et al. (2011). To further validate the downregulation of LRP1, we used anti-LRP1 primary antibody (1:10,000, Abcam, Catalog No. ab92544; RRID: AB_2234877) as well as β-actin (1:10,000; Sigma-Aldrich, Catalog No. A2228; RRID: AB_476697) as a control for normalization. The protein expression was visualized with the Clarity^TM^ Western ECL Substrate by BioRad (Feldkirchen, Germany, Catalog No. 1705061) and detected with the MicroChemie chemiluminescence device with Gel Capture Software by biostep (Burkhardtsdorf, Germany, Version 6.6).

### Cytokine Array

Hippocampal neurons were either co-cultured with wild-type or LRP1-deficient astrocytes for 21 days *in vitro*. Afterward, the cell culture supernatants were centrifuged for 10 min at 16,000 relative centrifugal force (rcf) to remove particles. Cytokine screening was performed with the Proteome Profiler^TM^ Mouse Cytokine Array Panel A (see Table 2; R&D Systems, Minneapolis, Minnesota, United States, Catalog No. ARY006).

**TABLE 2:** Coordinates of the 40 analyzed cytokines on the filter membrane of the assay.

Coordinate	Target cytokine
A1, A2	Reference spot
A3, A4	Reference spot
B1, B2	BLC
B3, B4	C5/C5a
B5, B6	G-CSF
B7, B8	GM-CSF
B9, B10	I-309
B11, B12	Eotaxin
B13, B14	sICAM-1
B15, B16	IFN-γ
B17, B18	IL-1α
B19, B20	IL-1β
B21, B22	IL-1ra
B23, B24	IL-2
C1, C2	IL-3
C3, C4	IL-4
C5, C6	IL-5
C7, C8	IL-6
C9, C10	IL-7
C11, C12	IL-10
C13, C14	IL-13
C15, C16	IL-12 p70
C17, 18	IL-16
C19, C20	IL-17
C21, C22	IL-23
C23, C24	IL-27
D1, D2	IP-10
D3, D4	I-TAC
D5, D6	KC
D7, D8	M-CSF
D9, D10	JE
D11, D12	MCP-5
D13, D14	MIG
D15, D16	MIP-1α
D17, D18	MIP-1β
D19, D20	MIP-2
D21, D22	RANTES
D23, D24	SDF-1
E1, E2	TARC
E3, E4	TIMP-1
E5, E6	TNF-α
E7, E8	TREM-1
F1, F2	Reference spot
F23, F24	Negative control

*Each coordinate represents an analyzed cytokine. The reference spots were positive controls, detectable after the application of the supernatant.*

### Statistical Analyses

Immunocytochemical stainings were quantified with ImageJ. All positive cells were counted with the cell counter plug-in. To investigate the influence of LRP1 on the differentiation of astrocytes, we quantified the number of tdTomato- and marker-double positive cells to compare the recombined subpopulations in both conditions. For the analysis of the pre- and postsynaptic puncta, the puncta analyzer with a rolling ball radius of 50 pixel was used. The size of a single puncta was defined as size (pixel^2^) = 2-infinity. The intensity of the bands of the PCR and Western blot analyses as well as the pixel densities of the cytokine array were evaluated with ImageJ. The band intensities were normalized to the expression of β-actin in both cases. We used the endpoint-PCR analysis as a semi-quantitative method to investigate differentially expressed genes upon LRP1 deletion. The number of cycles was below the level of amplificate saturation. The represented figures are exemplary pictures. The shown PCR results were repeated for representation and do not belong to the data shown in the diagram. Results are shown as the mean ± the standard error of the mean (SEM). After confirmation that the data were normally distributed, two-way ANOVA with *post hoc* Bonferroni test was performed to investigate the level of significance (*p* ≤ 0.05). If the data were not normally distributed, we used the Friedman test to assess the level of significance.

## Results

### Knockout Induction Was Successful in Astrocytic Cultures

**FIGURE 1:**
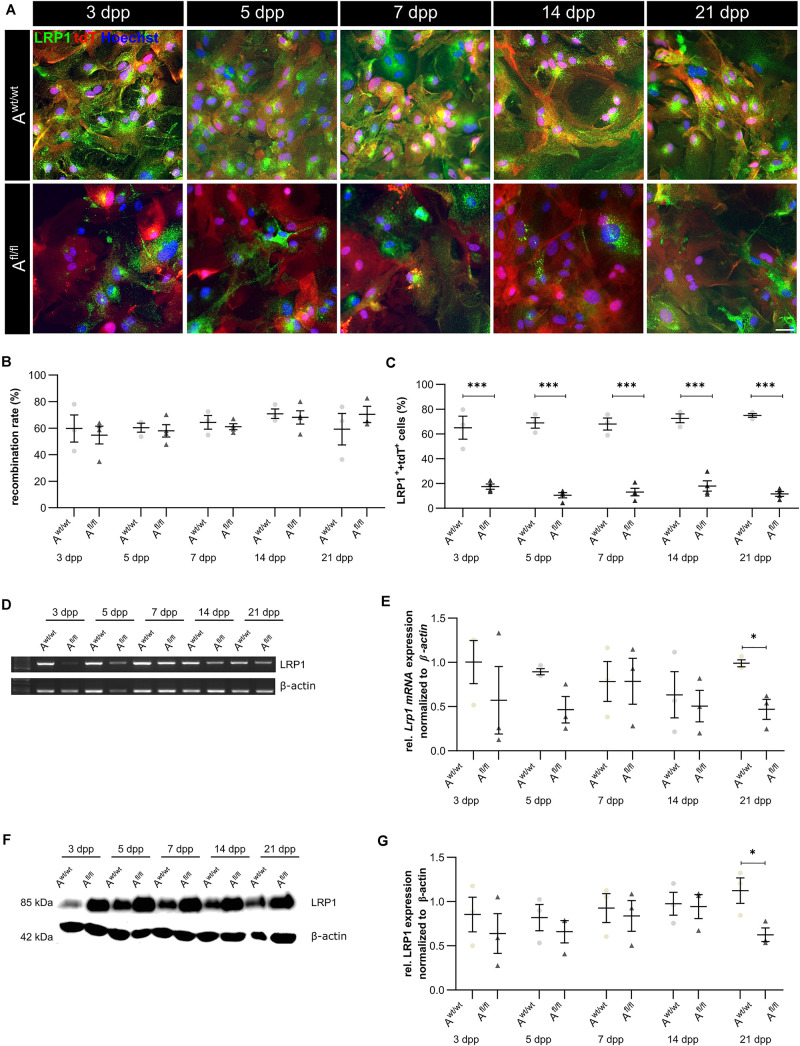
Knockout induction was successful *in vitro*. Immunocytochemical staining against LRP1 (green) and tdTomato (red) showed a decreased expression of LRP1 in recombined knockout astrocytes in comparison to recombined control astrocytes during the whole cultivation time **(A)**. The recombination rate of both conditions was similar, ranging between 60 and 70% of all astrocytes **(B)**. The deletion of *Lrp1* was assessed by the quantification of LRP1- and tdTomato-expressing cells **(C)**. The statistical evaluation showed that the number of LRP1-positive cells was significantly decreased in the knockout condition. Furthermore, the deletion of *Lrp1* was evaluated via PCR and compared to the expression in control astrocytes during the cultivation time up to 21 dpp **(D)**. The quantification revealed a significant downregulation of *Lrp1* in knockout astrocytes **(E)**. Additionally, the protein expression of LRP1 was significantly downregulated in knockout astrocytes in comparison to control astrocytes **(F,G)**. The PCR and Western blot analysis depict exemplary results (scale bar: 100 μm; mean ± SEM; *N* = 3; two-way ANOVA with *post hoc* Bonferroni test). *p < 0.05; ***p < 0.001.

We have previously shown that the deletion of *Lrp1* in NSPCs leads to an increased number of GFAP-positive astrocytes *in vitro* (Safina et al., 2016). To further validate the functions of LRP1 in astrocytes, we generated a new transgenic mouse line, where LRP1 can specifically be deleted from astrocytes via the expression of the Cre recombinase under the control of the GLAST promotor. Our control cells were lacking floxed LRP1, but expressing CreERT2 leading to recombined tdTomato expression after control cells were also treated with 4-OH-TAM. The recombination events were visualized via the reporter gene *Tdtomato* (Figure 1A). For this purpose, primary astrocytic cultures were treated exogenously with 4-hydroxy-tamoxifen (4-OH-TAM) to induce the activation of the Cre DNA recombinase. The recombination rate was evaluated via the quantification of all tdTomato-positive cells divided by the number of Hoechst-positive cell nuclei. The knockout efficiency was calculated as the number of LRP1- and tdTomato-double positive cells divided by the total number of tdTomato-positive cells. During the cultivation time from three up to 21 dpp, no differences in the recombination rates of control and knockout cells could be identified (Figure 1B). The expression of LRP1 in recombined cells was investigated in both conditions over the cultivation period (Figure 1C) to document the deletion of *Lrp1* in the knockout cultures. The statistical evaluation showed that the number of LRP1-positive cells was significantly decreased in the knockout condition compared to the control condition (3 dpp: A^wt/wt^ 65.05 ± 7.59%, A^fl/fl^ 17.47 ± 1.81%, *p* ≤ 0.0001; 5 dpp: A^wt/wt^ 68.88 ± 3.46%, A^fl/fl^ 10.51 ± 1.86%, *p* ≤ 0.0001; 7 dpp: A^wt/wt^ 68.01 ± 4.00%, A^f*l*/fl^ 13.04 ± 2.62%, *p* ≤ 0.0001; 14 dpp: A^wt/wt^ 72.60 ± 2.95%, A^fl/fl^ 17.96 ± 3.62%, *p* ≤ 0.0001; 21 dpp: A^wt/wt^ 74.99 ± 1.25%, A^fl/fl^ 11.61 ± 1.68%, *p* ≤ 0.0001). To further validate the downregulation of LRP1 in the astrocyte culture after induction with 4-OH-TAM, the expression of *Lrp1* on mRNA level was analyzed. Here, a decreased expression of *Lrp1* was observed in the knockout culture compared to the control culture. This effect was stable over the whole cultivation time and significant at 21 dpp (A^wt/wt^ 1.007 ± 0.028, A^fl/fl^ 0.468 ± 0.072 *p* ≤ 0.05; *N* = 3; Figures 1D,E). The quantification of the Western blot showed a decreased expression of LRP1 on the protein level in the knockout cultures during the whole cultivation time in comparison to the control condition. After 21 dpp, there was a significant decrease of astrocytic LRP1 in the depleted condition (0.625 ± 0.049) compared to the control (1.124 ± 0.144; *p* ≤ 0.05; *N* = 3) (Figures 1F,G). It is known that the knockout or downregulation of one member of the LDL family can cause increased expression of other members. Therefore, the expression of *Lrp2* and *Lrp4* was measured after the induction of the knockout. There were no alterations in the expression pattern of both receptors after the treatment with 4-OH-TAM in the LRP1-depleted cultures compared to the control condition (data not shown).

### LRP1 Was Not Implicated in the Proliferation of Early Postnatal Astrocytes

**FIGURE 2:**
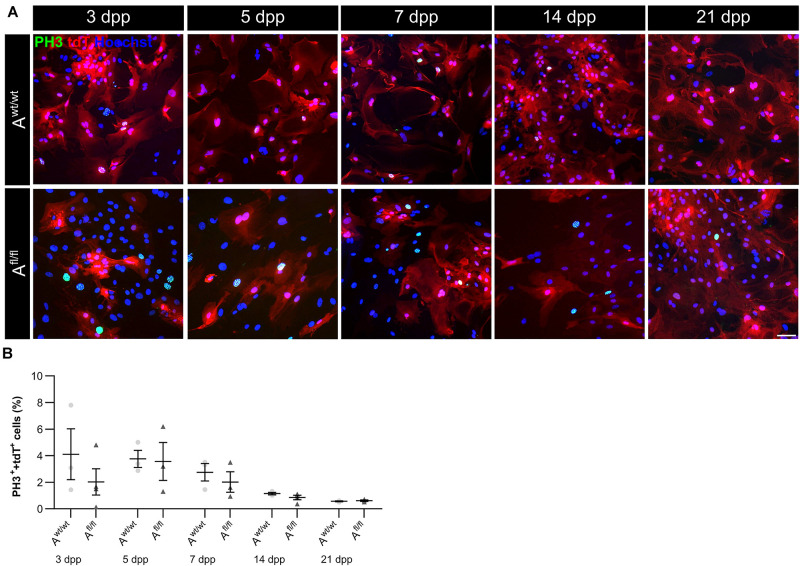
Proliferation rate was not significantly altered upon *Lrp1* deletion. The proliferation rate was evaluated with the marker phospho-histone H3 (PH3; green) and tdTomato (red) **(A)**. The quantification showed that with ongoing cultivation time, the proliferation rate was similar in both conditions **(B)** (scale bar: 100 μm; mean ± SEM; *N* = 3; two-way ANOVA with *post hoc* Bonferroni test).

Due to the high number of potential ligands and the influence of LRP1 on cellular processes, we investigated the proliferation capacity of astrocytes lacking LRP1. Therefore, we used the proliferation marker phospho-Histone H3 (PH3) (Figure 2A). At the beginning of the culture period, the immunocytochemical staining did not reveal significant differences between PH3- and tdTomato-double positive cells in LRP1-deficient astrocytes in comparison to control astrocytes. With ongoing differentiation, the number of proliferative cells decreased under both conditions (Figure 2B).

### Astrocyte Maturation Was Altered in Absence of LRP1

**FIGURE 3:**
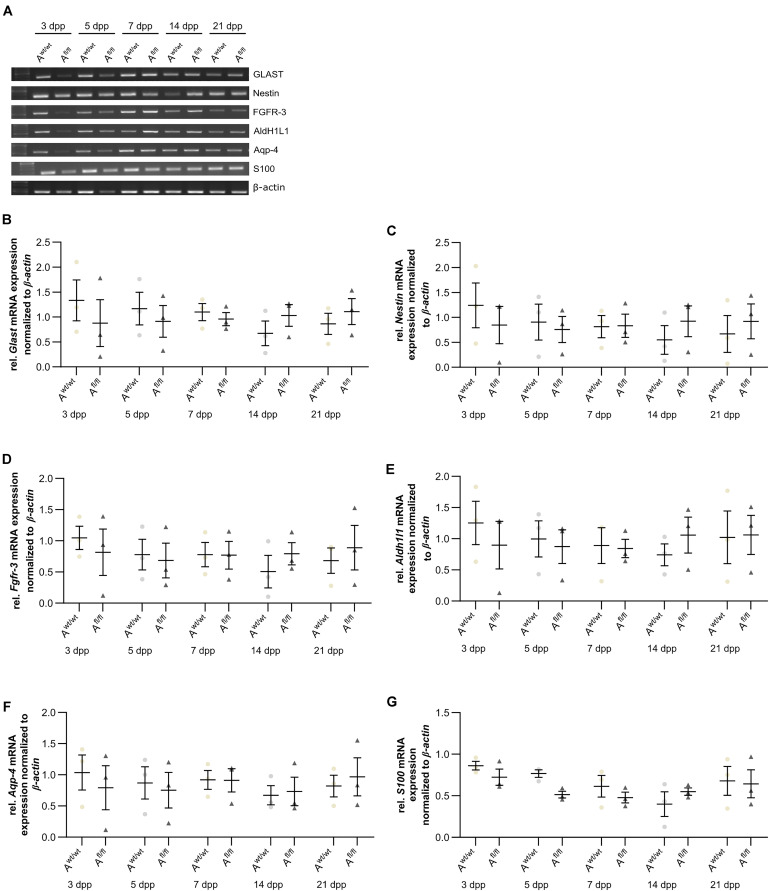
Expression of late precursor and immature genes was slightly altered at the beginning of the cultivation time in LRP1-deficient astrocytes. The expression of *Glast* was slightly decreased in LRP1-deficient astrocytes at 3 and 5 dpp **(A)**. With ongoing cultivation time, the expression increased in LRP1-depleted astrocytes and was comparable to control astrocytes **(B)**. The expression of *Nestin* showed a similar result **(A)**. First, the expression was slightly decreased in LRP1-knockout astrocytes and with ongoing maturation the amount increased and showed similar levels to control astrocytes **(C)**. As a last precursor gene, *Fgfr-3* was investigated **(A,D)**. Again, the expression was slightly decreased in depleted astrocytes, but with ongoing cultivation, the amount of *Fgfr-3* was increasing. The expression of *Aldh1l1* was slightly decreased in LRP1-depleted astrocytes 3 dpp compared to control astrocytes **(A,E)**. However, with ongoing maturation of the astrocytes, the amount of *Aldh1l1* was comparable between both genotypes. At the end of the cultivation time, the expression of *Aldh1l1* was slightly enhanced in LRP1-depleted astrocytes. The expression profile of *Aqp-4* was similar to the profile of *Aldh1l1* **(A,F)**. In immature cultures, the amount of *Aqp-4* was slightly decreased in knockout astrocytes and on a similar level with ongoing cultivation. The gene expression of *S100* was similar in LRP1-deficient astrocytes as well as in control astrocytes during the whole cultivation time **(A,G)**. The PCR analysis depicts exemplary results (mean ± SEM; *N* = 3; two-way ANOVA with *post hoc* Bonferroni test).

Next, the question was addressed if the deletion of *Lrp1* can cause an altered maturation of astrocytes. Therefore, a gene expression profile analysis was performed. We used stage-specific markers of the astrocytic maturation to determine the effect of LRP1 on the differentiation via RT-PCR. At first, late precursor cell genes were investigated. The expression of *Glast* was decreased at an early cultivation time in LRP1-deficient cultures in comparison to the control condition. With ongoing cultivation time, this effect was compensated and both conditions showed a similar expression of *Glast*. At the end of the cultivation time, LRP1-deficient astrocytes showed an increased expression of *Glast* in comparison to the control condition (Figures 3A,B). Next, the expression of the late precursor gene *Nestin* was analyzed (Figures 3A,C). Here, the PCR revealed a similar effect of LRP1 on the *Nestin* expression as on the *Glast* expression. At the beginning of the cultivation, LRP1-depleted astrocytes expressed a decreased amount of *Nestin* in comparison to the control astrocytes. After a longer period of time, the expression profile was adapted in both conditions. After 14 and 21 dpp, the expression of *Nestin* was slightly enhanced in LRP1-deficient astrocytes compared to control astrocytes (Figure 3C). Afterward, the levels of the *fibroblast growth factor receptor-3* (FGFR-3) were analyzed as a marker for late precursor cells, which will eventually differentiate into astrocytes (Figure 3A). At early cultivation timepoints, control astrocytes showed an increased *Fgfr-3* expression compared to LRP1-depleted astrocytes. The absence of LRP1 in the astrocytes caused slightly increased levels of *Fgfr-3* after a longer cultivation time in comparison to the control condition (Figure 3D).

Immature astrocytes are characterized by the expression of aquaporin-4 (Aqp-4) as well as aldehyde dehydrogenase 1 family member L1 (AldH1L1). The expression of *Aldh1l1* was slightly decreased in LRP1-deficient astrocytes at an early timepoint compared to control astrocytes. With ongoing differentiation, the gene expression increased in knockout astrocytes and was similar to the amount of *Aldh1l1* in control astrocytes (Figures 3A,E). At the beginning of the cultivation time, *Aqp-4* was slightly decreased in LRP1-deficient astrocytes in comparison to control astrocytes. One week after plating, the amount of *Aqp-4* was similar in the control and knockout condition. With increasing cultivation time, the expression of *Aqp-4* increased in LRP1-lacking astrocytes in comparison to control astrocytes (Figures 3A,F).

To evaluate the differentiation from LRP1-deficient astrocytes into mature astrocytes, we used the S100 calcium binding protein (S100). Here, the analysis revealed no alterations in the amount of *S100* on mRNA level in knockout astrocytes compared to the control group (Figures 3A,G).

The analysis revealed that late precursor and immature genes showed the same shift in LRP1-deficient astrocytes. At early cultivation timepoints, LRP1-knockout astrocytes showed a decreased expression of these genes in comparison to control astrocytes. However, with ongoing differentiation, LRP1-deficient astrocytes compensated the decreased expression and showed an increased amount of gene expression compared to the control condition. In conclusion, we observed a shifted expression pattern of late precursor and immature astrocytic genes in LRP1-depleted astrocytes.

### LRP1-Deficient Astrocytes Did Not Show a Reactive Phenotype

**FIGURE 4:**
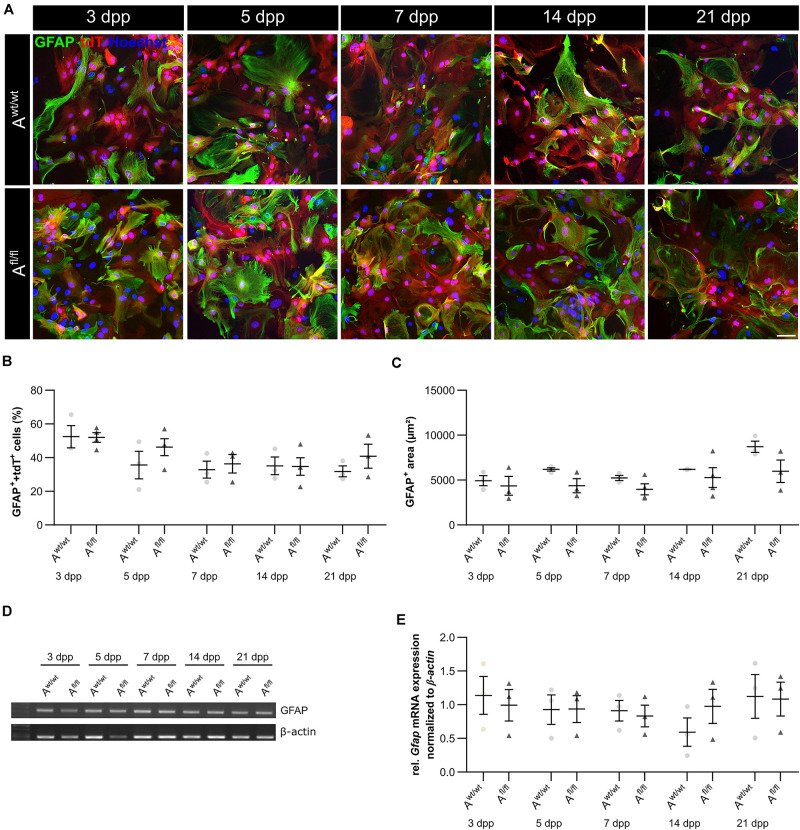
LRP1-deficient astrocytes showed no reactive phenotype. The immunocytochemical staining against GFAP (green) and tdTomato (red) revealed no alterations in the morphology of astrocytes **(A)**. The quantification of the staining showed no changes in the number of GFAP- and tdTomato-double positive astrocytes in the LRP1-depleted condition compared to the control condition **(B)**. Reactive astrocytes showed an increased GFAP-positive area compared to non-reactive astrocytes. However, the GFAP-positive area was similar in both conditions and showed no changes **(C)**. Additionally, the gene expression of *Gfap* was investigated **(D)**. The statistical evaluation showed no alterations in both conditions **(E)**. The PCR analysis depicts exemplary results (scale bar: 100 μm; mean ± SEM; *N* = 3; two-way ANOVA with *post hoc* Bonferroni test).

It is known that astrocytes react to stress with an increased expression of GFAP. Besides an increased number of GFAP-positive cells, the area of the astrocytes can increase as well. Therefore, we were interested in the number of GFAP-positive cells in the absence of LRP1, as well as in the GFAP-positive area covered by these astrocytes. The immunocytochemical staining did not reveal any changes in the number of GFAP- and tdTomato-double positive astrocytes in LRP1-lacking cultures compared to the control condition (Figure 4B). The measurement of the area covered by GFAP-positive astrocytes was not increased in the absence of LRP1 compared to control astrocytes (Figure 4C). The evaluation of the expression of *Gfap* mRNA via PCR revealed the same tendency. The ablation of LRP1 caused no alterations in comparison to the control condition (Figures 4D,E). The deletion of LRP1 did not cause a reactive phenotype in regard to the number of GFAP-positive cells as well as on the area covered by GFAP-positive astrocytes.

### LRP1-Deletion Resulted in Increased Number of the Glutamate Transporter 1-Positive Astrocytes (GLT-1)

**FIGURE 5:**
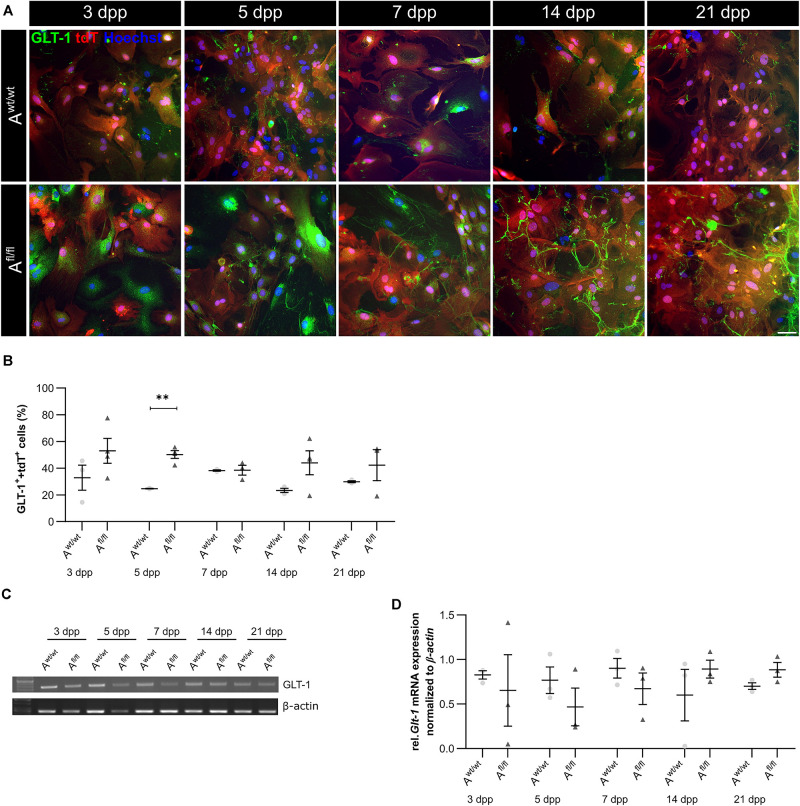
*Lrp1* deletion resulted in increased number of GLT-1-positive astrocytes. The number of GLT-1 (green)- and tdTomato (red)-double positive astrocytes was evaluated via immunocytochemistry **(A)**. The quantification showed a significantly increased number of GLT-1- and tdTomato-double positive astrocytes upon LRP1 deletion. With ongoing cultivation time, the number of double-positive cells decreased slightly in the LRP1-depleted culture, but remained higher than in the control condition **(B)**. Additionally, the gene expression of *Glt-1* was investigated **(C)** but the quantification revealed no changes between both conditions **(D)**. The PCR analysis depicts exemplary results (scale bar: 100 μm; mean ± SEM; *N* = 3; two-way ANOVA with *post hoc* Bonferroni test). **p < 0.01.

Beside various functions, astrocytes can also influence synaptic transmission via the uptake of neurotransmitters such as glutamate out of the synaptic cleft. One glutamate transporter in mature astrocytes is the glutamate transporter 1 (GLT-1). We investigated the number of GLT-1-positive astrocytes via immunocytochemistry and the amount of *Glt-1* expression via PCR analysis. Here, we observed an increased number of GLT-1-positive astrocytes in the absence of LRP1 in comparison to control astrocytes (Figures 5A,B). The difference between the knockout and control situation was more pronounced in the beginning of the cultivation time. At 5 dpp, the number of GLT-1- and tdTomato-double positive astrocytes were significantly increased in LRP1-depleted astrocytes (50.27 ± 2.20%) compared to control astrocytes (24.835 ± 0.09%; *p* ≤ 0.05; *N* = 3). With increasing cultivation, the number of GLT-1- and tdTomato-double positive astrocytes was slightly diminished in control cultures compared to LRP1-knockout astrocytes. However, the gene expression of *Glt-1* was not altered in LRP1-depleted astrocytes in contrast to control astrocytes (Figures 5C,D). The deletion of LRP1 in astrocytic cultures resulted in an increased number of GLT-1-positive astrocytes. Because the control and the LRP1-deficient astrocytes have the same genetic background as a consequence of our breeding strategy, the phenotype observed can directly be traced back to the receptor and most probably implicates LRP1-coupled signaling pathways.

### LRP1-Deficient Astrocytes Promote the Generation of Presynapses and Decreased the Activity of Hippocampal Neurons

**FIGURE 6:**
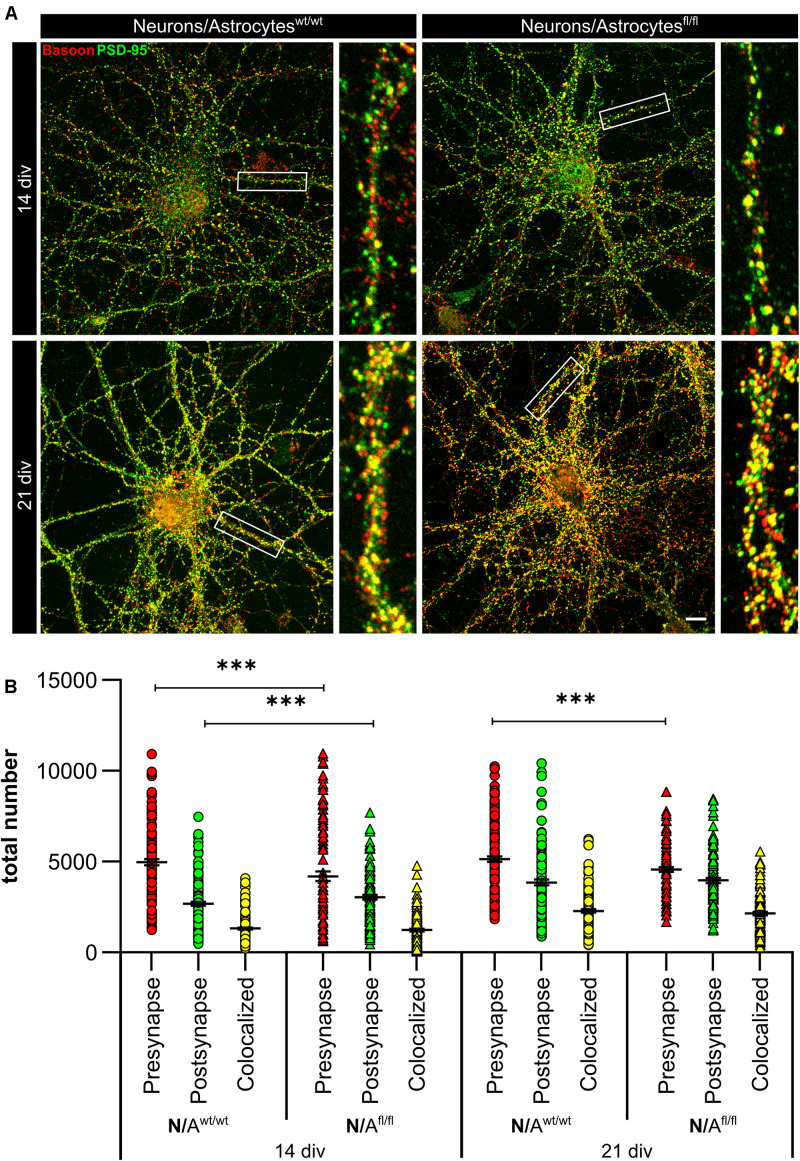
*Lrp1* deletion in astrocytes altered neuronal synaptogenesis significantly. Hippocampal neurons (N) were either co-cultured with wild-type (N/A^wt/wt^) or LRP1-depleted (N/A^fl/fl^) astrocytes for 14 and 21 div to evaluate the number of presynapses (Bassoon, red), postsynapses (PSD-95, green), and structurally complete synapses (colocalization, yellow) **(A)**. The quantification showed that the number of presynapses was significantly decreased in N/A^fl/fl^ compared to N/A^wt/wt^ during the whole cultivation time. Additionally, the number of postsynapses was significantly increased in N/A^fl/fl^ at 14 div compared to N/A^wt/wt^. However, the amount of structurally complete synapses was not influenced by the LRP1-depleted astrocytes in comparison to the control condition **(B)** (scale bar: 100 μm; mean ± SEM; N/A^wt/wt^ *N* = 6, *n* = 151; N/A^fl/fl^ *N* = 5, *n* = 130; two-way ANOVA with *post hoc* Bonferroni test). ***p < 0.001.

**FIGURE 7:**
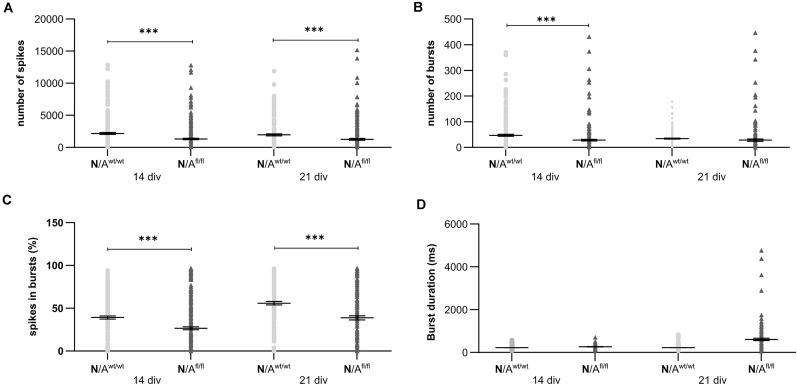
Neuronal activity was significantly altered in neurons co-cultured with LRP1-deficient astrocytes. The number of spikes is defined as spontaneous single action potentials. N/A^fl/fl^ showed a significantly decreased number of spikes after 14 and 21 div compared to N/A^wt/wt^ **(A)**. Additionally, the number of bursts, which is the cumulated sequence of several action potentials, was significantly decreased in N/A^fl/fl^ compared to the control condition at 14 and 21 div **(B)**. Another investigated parameter was the percentage of spikes in bursts. The percentage was significantly decreased at both analyzed timepoints in N/A^fl/fl^ compared to N/A^wt/wt^ **(C)**. Also, the duration of bursts was measured. At 14 div, the duration was similar in both conditions. However, at 21 div, the burst duration was increased in N/A^fl/fl^ compared to N/A^wt/wt^ **(D)** (mean ± SEM; *N* = 4, *n* = 240; two-way ANOVA with *post hoc* Bonferroni test). ***p < 0.001.

To further elucidate the influence of LRP1 on the functionality of astrocytes, we co-cultured embryonic wild-type hippocampal neurons (further referred to as N) either with wild-type astrocytes (N/A^wt/wt^) or with LRP1-deficient astrocytes (N/A^fl/fl^). The synaptogenesis was investigated with immunocytochemical stainings using the presynapse marker Bassoon and the postsynaptic marker PSD-95 (Figure 6A). The colocalization of both signals was pictured in yellow puncta, highlighting a structurally complete synapse. The total number of pre- as well as postsynapses and the number of structurally complete synapses were quantified after 14 and 21 days *in vitro* (div). After 14 div, the number of presynapses was significantly decreased in N/A^fl/fl^ (4188.938 ± 261.4586) when compared with N/A^wt/wt^ (4969.669 ± 171.561; *p* ≤ 0.05; *N* = 5; *n* = 130). In contrast, the number of postsynapses was significantly increased in N/A^fl/fl^ (3035.577 ± 131.638) compared to N/A^wt/wt^ (2676.801 ± 105.416; *p* ≤ 0.05; *N* = 5; *n* = 130). However, the number of structurally complete synapses as indicated by the number of yellow puncta revealed no changes in both conditions after 14 div. After 21 div, the number of Bassoon-positive puncta was again significantly decreased in N/A^fl/fl^ (4567.228 ± 129.309) when compared to N/A^wt/wt^ (5137.953 ± 151.525; *p* ≤ 0.05; *N* = 5; *n* = 130). However, the number of postsynapses as well as of colocalization was not altered in N/A^fl/fl^ in respect to N/A^wt/wt^ (Figure 6B). The neuronal activity of neurons co-cultured with either wild-type or LRP1-deficient astrocytes was evaluated with a MEA. The investigated parameters were the number of spikes, defined as spontaneous single action potential, as well as bursts, an organized sequence of several action potentials, and the percentage of spikes in bursts as well as the duration of bursts. These parameters are highlighting the neuronal activity. After 14 div, the number of spikes was significantly decreased in N/A^fl/fl^ (1308.387 ± 110.463) compared to N/A^wt/wt^ (2168.304 ± 161.824; *p* ≤ 0.0001; *N* = 4; *n* = 240) (Figure 7A). Moreover, this effect was visible with increasing cultivation time, where the number of spikes was significantly decreased in N/A^fl/fl^ (1259.662 ± 137.21) when compared to N/A^wt/wt^ (1951.156 ± 153.940; *p* ≤ 0.001; *N* = 4; *n* = 240). The organized activation of neurons, defined as bursts, was significantly decreased in N/A^fl/fl^ (28.334 ± 4.55) in contrast to N/A^wt/wt^ (47.217 ± 3.739; *p* ≤ 0.0001; *N* = 4; *n* = 240) after 14 div. Furthermore, the number of bursts remained similar in N/A^fl/fl^ after 21 div and was slightly decreased compared to N/A^wt/wt^ (Figure 7B). The percentage of spikes in bursts highlights the organization of the activity of the whole neuronal network. Here, the measurements showed that the co-culture of LRP1-deficient astrocytes together with hippocampal neurons resulted in a significantly decreased percentage of spikes in bursts over the whole cultivation time (14 div: 26.60 ± 1.93%; 21 div: 38.79 ± 2.52%), when compared to neurons co-cultured with wild-type astrocytes (14 div: 39.22 ± 1.86%; 21 div: 55.92 ± 2.04%; *p* ≤ 0.0001; *N* = 4; *n* = 240) (Figure 7C). At the first investigated timepoint, the burst duration was comparable in N/A^wt/wt^ and N/A^fl/fl^. After 21 div, the burst duration was increased in N/A^fl/fl^ in contrast to N/A^wt/wt^ (Figure 7D). The deletion of astrocytic LRP1 resulted in a decreased number of single action potential and number of bursts in hippocampal neurons as well as in an altered synaptogenesis. This result was more remarkable because the N/A^fl/fl^ culture system contained a minor fraction of wild-type astrocytes (section “Knockout Induction Was Successful in Astrocytic Cultures”, see above), which may partially alleviate the impact of LRP1 elimination. These findings highlight the importance of astrocytic LRP1 in regard to the directed neuronal network activity *in vitro*.

### Astrocytic *Lrp1* Deletion Did Not Cause Altered Glutamate Transporter and Glutamate Receptor Expression

**FIGURE 8:**
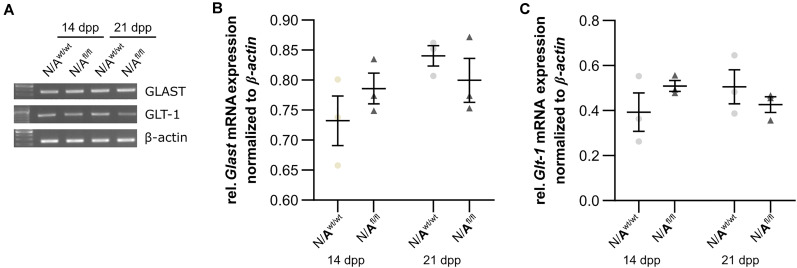
LRP1 did not alter astrocytic functions in regard to glutamate transport. Both astrocytic glutamate transporters were investigated in either LRP1-deficient or wild-type astrocytes co-cultured with hippocampal neurons. The gene expression profile of *Glast* was comparable in N/A^wt/wt^ and N/A^fl/fl^ at both timepoints **(A,B)**. Moreover, the relative expression of *Glt-1* was also not altered in N/A^wt/wt^ compared to N/A^fl/fl^ **(A,C)**. The PCR analysis depicts exemplary results (mean ± SEM; *N* = 3; two-way ANOVA with *post hoc* Bonferroni test).

To further investigate the molecular influence of LRP1 on astrocytic functions in regard to neuronal transmission, we analyzed the expression of both glutamate transporter GLAST and GLT-1 in either LRP1-depleted or control astrocytes co-cultured with hippocampal neurons for 14 and 21 div. Here, the expression pattern of *Glast* was not altered on mRNA level in the knockout condition when compared to wild-type astrocytes co-cultured with neurons (Figures 8A,B). Also, the expression of *Glt-1* was similar in both conditions after 14 and 21 div (Figures 8A,C).

**FIGURE 9:**
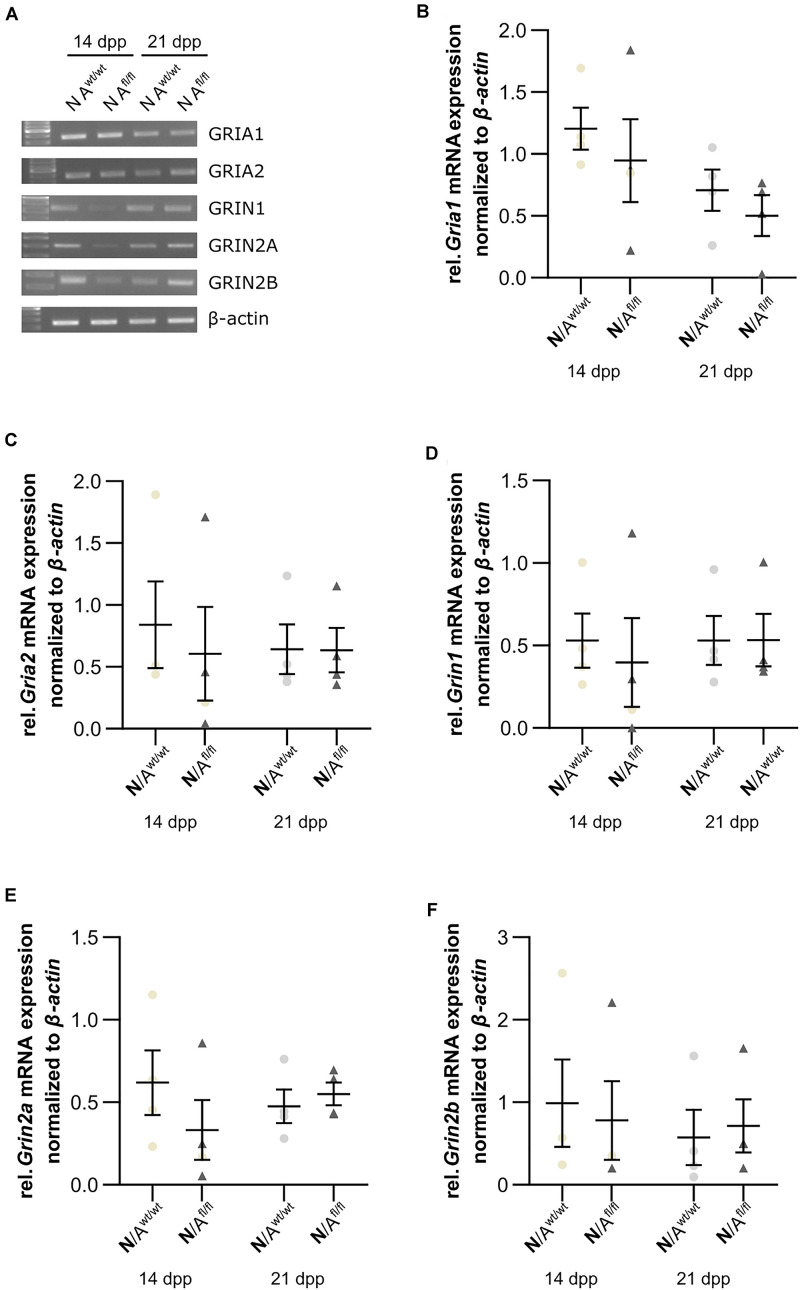
Glutamate receptor subunits were not influenced by astrocytic LRP1 deletion. **(A)** The AMPA receptor subunits *Gria1* and *Gria2* showed no changes in the gene expression profile of N co-cultured either with A^wt/wt^ or with A^fl/fl^ at 14 or 21 div **(B,C)**. Additionally, the expression profile of the obligatory NDMA receptor subunit *Grin1* was investigated and again the absence of astrocytic LRP1 had no influence of the amount of *Grin1* **(D)**. Moreover, two NMDA receptor subunits were investigated, which are known to be influenced by LRP1. However, the amount of *Grin2a* and *Grin2b* were not significantly influenced by astrocytic LRP1 **(E,F)**. The PCR analysis depicts exemplary results (mean ± SEM; *N* = 4; Friedman test).

Next, we were interested in the expression profile of glutamate receptor subunits of the AMPA and NMDA receptors (AMPAR and NDMAR). Therefore, we performed a mRNA analysis of hippocampal neurons, which were co-cultured with either wild-type or LRP1-depleted astrocytes for 14 and 21 div. The analysis revealed no alterations in the expression of the analyzed AMPAR subunits *Gria1* and *Gria2* in neurons co-cultured with wild-type astrocytes after 14 and 21 div (Figures 9A,B and Figures 9A,C), when compared to neurons co-cultured with LRP1-deficient astrocytes. Next, we investigated the obligatory subunit *Grin1* of the NMDAR. Again, the expression profile of the neurons was not influenced by LRP1-depleted astrocytes after 14 and 21 div when compared to the neurons co-cultured with wild-type astrocyte (Figures 9A,D). The relative expression of the alternative NMDAR subunit *Grin2a* was comparable in neurons co-cultured with wild-type or with LRP1-deficient astrocytes (Figures 9A,E). The relative expression of *Grin2b* was slightly increased at 14 div in neurons co-cultured with wild-type astrocytes, whereas the relative expression in neurons co-cultured with LRP1-deficient astrocytes was slightly decreased. Nevertheless, the alterations in the relative expression of *Grin2b* after 21 div were not detectable anymore (Figures 9A,F). In conclusion, the decreased neuronal network activity of hippocampal neurons co-cultured with LRP1-deficient astrocytes was caused by neither the altered expression of NMDAR- or AMPAR-subunits in neurons nor the expression of glutamate transporters on the astrocytic cell surface.

### LRP1-Deficient Astrocytes Showed no A1 Astrocyte-Specific Phenotype

**FIGURE 10:**
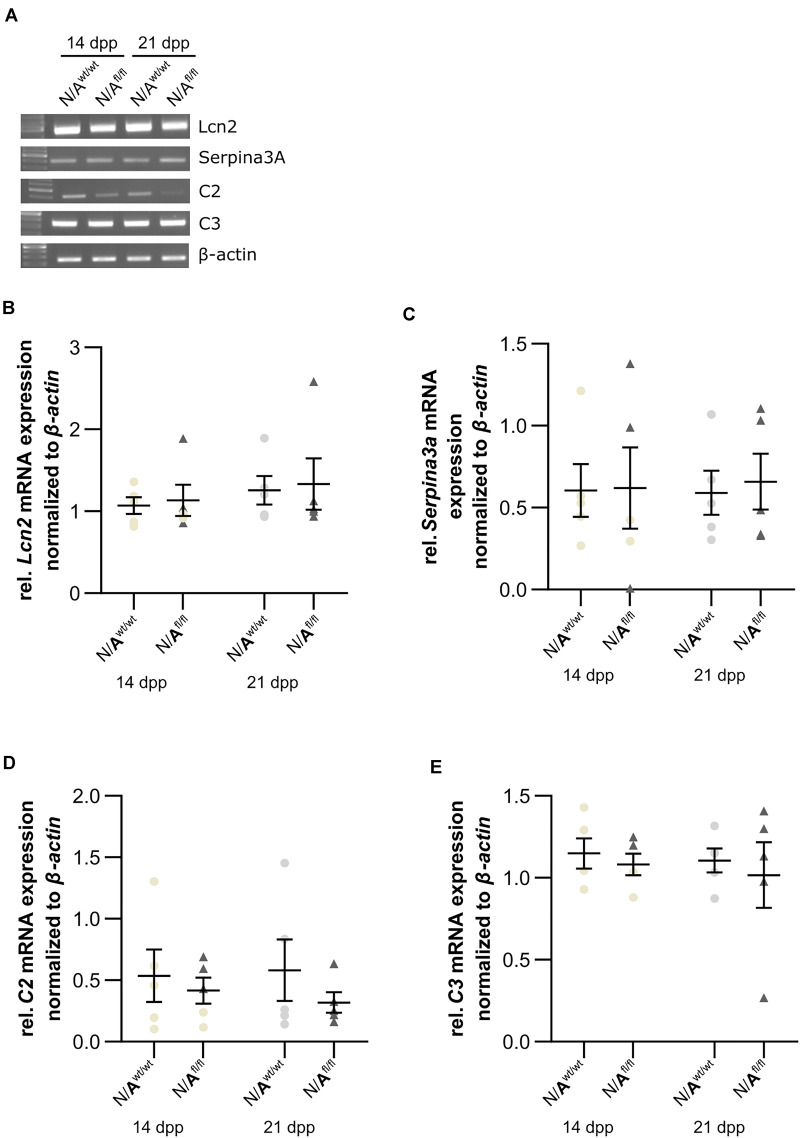
LRP1-deficient astrocytes did not express neurotoxic factors. The relative expression of *lipocalin-2* (*Lcn2*) was not altered in N/A^fl/fl^ compared to N/A^wt/wt^ **(A,B)**. Another factor secreted by neurotoxic astrocytes is the serine protease inhibitor A3A (Serpina3A) **(C)**. The gene expression profile was not altered in N/A^fl/fl^ compared to N/A^wt/wt^ during the cultivation time. Additionally, two components of the complement system were analyzed **(D,E)**. Again, both components, *C2* and *C3*, were similarly expressed in both conditions. The PCR analysis depicts exemplary results (mean ± SEM; *N* = 5; Friedman test).

To further validate whether the deletion of LRP1 in astrocytes caused neurotoxic properties, we investigated the expression of factors that are known to be expressed by type A1 astrocytes (Clarke et al., 2018). Therefore, we used lysates of wild-type or LRP1-deficient astrocytes that had been co-cultured with hippocampal neurons for 14 and 21 div. Firstly, the expression profile of *lipocalin-2* (*Lcn2*) was analyzed. *Lcn2* expression was not altered in LRP1-deficient astrocytes when compared to wild-type astrocytes (Figures 10A,B). Another factor expressed by A1 astrocytes is the serine protease inhibitor A3A (Serpina3A). Here, the analysis revealed no alterations in the absence of astrocytic LRP1 in comparison to wild-type astrocytes co-cultured with neurons (Figures 10A,C). The over-activation of the complement system can have a negative impact on synaptogenesis and neuronal activity, which can be influenced by A1 astrocytes. Therefore, we also investigated the expression of the complement components C2 and C3, which are known to be highly upregulated in A1 astrocytes. The expression of *C2* was slightly decreased in LRP1-deficient astrocytes when compared to wild-type astrocytes (Figures 10A,D). Another component of the complement system that was analyzed was C3. During the whole cultivation time, the deletion of LRP1 had no influence on the relative expression of *C3*, when compared to the wild-type situation (Figures 10A,E). In summary, LRP1-depleted astrocytes did not show the expression pattern of neurotoxic A1 astrocytes.

### Expression of Cytokines Were Altered in Cell Culture Supernatant of LRP1-Deficient Astrocytes Co-Cultured With Hippocampal Neurons

**FIGURE 11:**
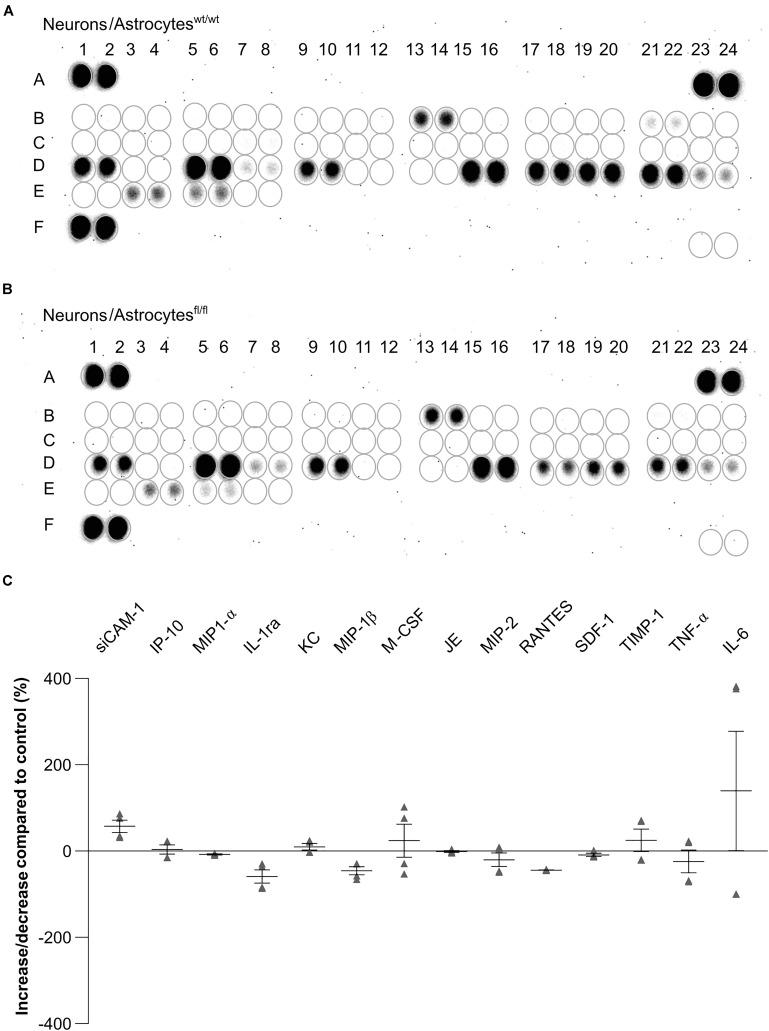
Expression of cytokines in the supernatant of hippocampal neurons co-cultured with wild-type **(A)** or LRP1-deficient astrocytes **(B)** after 21 div. The quantification revealed that the cytokines KC and TIMP-1 were slightly increased expressed in N/A^fl/fl^ compared to the control condition **(C)**. Additionally, siCAM-1 and IL-6 were strongly expressed in the knockout condition, whereas IP-10, MIP-1α, JE, MIP-2, and SDF-1 were slightly decreased in the knockout condition compared to the control. Strongly reduced were the cytokines IL-ra, MIP-1β, M-CSF, RANTES, and TNF-α (mean ± SEM; *N* = 2, *n* = 4).

To further investigate the influence of astrocytic LRP1 on synaptogenesis and neuronal activity, we were interested in the expression of cytokines in the supernatant of hippocampal neurons either co-cultured with wild-type or LRP1-deficient astrocytes after 21 div. Both conditions expressed the same cytokines, however, with differing amounts (Figures 11A,B). The analysis showed that the cytokines KC and TIMP-1 were slightly increased in N/A^fl/fl^ in comparison to other upregulated cytokines. A strong increased expression of siCAM-1 and IL-6 was observed in the knockout condition. The cytokines IP-10, MIP-1α, JE, and SDF-1 were slightly decreased in the supernatant of N/A^fl/fl^ compared to N/A^wt/wt^ (Figure 11C). However, some cytokines were strongly downregulated in the knockout condition. IL-ra showed a decreased expression in the knockout condition compared to the control condition. Furthermore, MIP-1β and M-CSF as well as MIP-2, RANTES, and TNFα were strongly downregulated upon deletion of *Lrp1* in astrocytes co-cultured with hippocampal neurons compared to the control condition (Figure 11C).

## Discussion

We generated an inducible conditional transgenic mouse model where *Lrp1* was specifically deleted in GLAST-positive astrocyte precursor cells. With this approach, we wanted to investigate the influence of LRP1 on the maturation and functionality in astrocytes. Additionally, we wanted to unravel the functions of astrocytic LRP1 on synaptogenesis and neuronal activity *in vitro*. Our data provide evidence that LRP1-deficient astrocytes caused a significantly altered synaptogenesis of hippocampal neurons as well as a decreased number of single action potentials and neuronal network activity.

Lipoprotein receptor-related protein 1 regulates the availability of the tPa at synapses via endocytosis and releases tPa in a controlled manner (Cassé et al., 2012). The inhibition of LRP1 via its inhibitor receptor related protein (RAP) reduced the amount of tPA uptake by astrocytes. However, the release of neuronal tPA was not influenced by a decreased astrocytic tPA uptake (Cassé et al., 2012). We hypothesize that the increased number of postsynapses in our experimental setup was caused by an increased amount of tPA in the synaptic cleft, because the endocytosis by LRP1-deficient astrocytes was impaired. As already described, one of the multiple ligands of LRP1 is ApoE, which is involved in cholesterol transport. Neurons alone are able to produce enough cholesterol to survive and differentiate to some extent, but their synapse formation is inefficient (Pfrieger, 2003). Neurons need to be supplied with cholesterol via astrocytes and ApoE-containing lipoproteins to form structural and functional synapses. Massive synaptogenesis requires large amounts of cholesterol, which may represent a link between synaptogenesis and the differentiation of astrocytes in the developing brain (Mauch et al., 2001). The study of Liu et al. (2010) showed that the deletion of *Lrp1* in the brain resulted in increased levels of ApoE but decreased levels of cholesterol. The availability of cholesterol can impair the development of presynapses in our co-culture system with LRP1-deficient astrocytes. We propose that the deletion of *Lrp1* in astrocytes co-cultured with hippocampal neurons caused an altered pre- and postsynapse formation due to modified uptake and degradation of tPA and ApoE. This presumably resulted in altered cholesterol availability for neurons and affected ongoing synaptogenesis.

The analysis of the neuronal activity revealed that the deletion of astrocytic LRP1 resulted in a decreased number of single action potential as well as a decreased network activity in general. Among other things, astrocytes remove glutamate out of the synaptic cleft via glutamate transporters, e.g., GLT-1, which is significantly increased in our *in vitro* model. Furthermore, they supply the presynaptic terminal with glutamate, as well as with the precursor glutamine, and astrocytes support the efficient uptake of glutamate following synaptic transmission (Papouin et al., 2017). Rehman et al. (2017) have shown that glutamine augments neuronal activity in rat hippocampal slices. Therefore, we conclude that the deletion of LRP1 in astrocytes might cause an altered supply of glutamine at the presynaptic terminal, which resulted in a decreased neuronal activity and weakened presynaptic terminals.

Additionally, we investigated if the maturation and differentiation of astrocytes were influenced by the deletion of LRP1. The analyses showed that LRP1-deficient astrocytes had a reduced proliferation rate at the beginning of the cultivation. This effect was already described in neural stem and progenitor cells (NSPCs), which were lacking LRP1 (Safina et al., 2016). It was reported that astrocytes *in vitro* showed a density-dependent proliferation (Nakatsuji and Miller, 1998), explaining the similar proliferation rates observed in our model at later cultivation timepoints. However, our findings suggest that the absence of LRP1 also has an impact on the proliferation of astrocytes *in vitro*.

The next focus was the influence of LRP1 on the maturation of astrocytes. Therefore, we used stage-specific markers to address this question. Here, we used several tools to investigate the impact. Firstly, we analyzed the expression of late precursor, immature, and mature astrocyte genes via PCR analysis. All investigated genes have in common that at the beginning of the cultivation time, the expression of these genes was decreased in the LRP1-depleted astrocytes in comparison to the control astrocytes. Subsequently, with ongoing differentiation, the expression of the genes was increased in the deficient cultures in comparison to the control. These findings lead to the suggestion that LRP1 might be important for the early-onset differentiation of astrocytes *in vitro*. This is supported by the results of Herz et al. (1992, 1993) where it was shown that the deletion of *Lrp1* in the embryo is lethal, highlighting the importance of LRP1 in the early development of the CNS. Additionally, the altered or decreased expression of stage-specific markers corresponds to the decreased proliferation rate observed in the LRP1-deficient astrocytes. A decreased proliferation rate might result in an altered or delayed differentiation of astrocytes. However, neither the number of GFAP-positive astrocyte nor the GFAP-positive area was altered in the absence of LRP1.

Astrocytes are known as the most abundant cell type in the CNS (Zuchero and Barres, 2015; Liddelow and Barres, 2017) and mediate diverse functions. They are involved in energy supply (Chuquet et al., 2010), maintenance of ion and pH homeostasis (Mahmoud et al., 2019), and the uptake of neurotransmitters, e.g., γ-aminobutyric acid (GABA), glycine, and glutamate. Thereby, astrocytes are also involved in the regulation of synaptic transmission (Renzel et al., 2013). The concept of the tripartite synapse, where astrocytes in conjunction with the pre- and postsynapse form the functional synapse, is a well-established model (Farhy-Tselnicker and Allen, 2018). Astrocytic protrusions, including those that contact synapses, express several proteins, which are involved in synaptic transmission (Papouin et al., 2017). For example, astrocytic protrusions express the GLAST and the glutamate transporter-1 (GLT-1) (Chaudhry et al., 1995), glutamine synthetase (Derouiche and Frotscher, 1991), aquaporins (Thrane et al., 2011), potassium channels (Higashi et al., 2001), cell adhesion molecules, and lactate transporters (Zhuang et al., 2011). These factors contribute to the controlled and effective neuronal transmission by resupplying the presynaptic terminal with glutamate and glutamate precursor glutamine via glutamate transporters and glutamine synthetase. Additionally, astrocytes are removing glutamate out of the synaptic cleft to ensure an efficient synaptic transmission. Lastly, they provide neurons with energy substrates to support the neuronal transmission, as well as plasticity (Papouin et al., 2017). An altered astrocyte functionality, e.g., a decreased expression of glutamate transporters on the cell surface, can cause pathological conditions. Regan et al. (2007) have reported that the decreased expression of glutamate transporters resulted in an increased amount of glutamate in the synaptic cleft. An increased amount of glutamate above the threshold is neurotoxic and causes hyperexcitability of neurons. The immunocytochemical staining against GLT-1 showed that the deletion of *Lrp1* caused an increased number of GLT-1-positive astrocytes in comparison to the control condition. Previous studies reported that the expression of GLT-1 or number of GLT-1-positive astrocytes decreased after the onset of seizures or *status epilepticus* (SE) (Hubbard et al., 2016). However, recently, Peterson and Binder (2019) have demonstrated that GLT-1 and GLAST, both glutamate transporters, were significantly upregulated shortly after SE but were decreased at later timepoints. They conclude that this was a compensatory response to the large amount of glutamate during SE. We already hypothesized that the altered neuronal activity was caused by an inefficient supply of glutamine at the presynaptic terminal and the decreased replenishment of glutamate via the LRP1-deficient astrocytes, which might result in an increased number of GLT-1-positive astrocytes.

Our recent data demonstrated that the proliferation rate was decreased in LRP1-depleted astrocytes, which resulted in a delayed maturation of the cells *in vitro*. Additionally, the number of GLT-1-positive astrocytes was significantly increased in the absence of LRP1.

To investigate the influence of astrocytic LRP1 on the functionality of neurons, we analyzed the gene expression of NMDA and AMPA receptor subunits in neurons co-cultured with LRP1-deficient or wild-type astrocytes, as previous studies reported that the activation of NMDA and AMPA receptors can be influenced by astrocytic properties (Xu et al., 2010; Allen, 2014; Jolly et al., 2018; Neame et al., 2019). However, we did not see an alteration in the expression of receptor subunits when neurons were co-cultured with LRP1-deficient astrocytes. Clarke et al. (2018) have shown that astrocyte reactivity is accompanied by the expression of specific genes. Therefore, we investigated if the deletion of *Lrp1* in astrocytes contributes to a reactive phenotype in the presence of hippocampal neurons, which might lead to the release of neurotoxic factors. Here we chose, in accordance with Clarke et al. (2018), lipocalin-2 (Lcn2), serine protease inhibitor A3A (Serpina3A), as well as the two complement components, C2 and C3. The quantification revealed no altered expression of any of these genes in LRP1-deficient astrocytes. This leads to the assumption that the absence of LRP1 had no influence on the reactivity of astrocytes on mRNA level. This is consistent with the observation that the number of GFAP-positive astrocytes and their surface area was not altered upon the deletion of *Lrp1*. So, we conclude that a reactive phenotype of the astrocytes is not the reason for the altered activity of the neurons co-cultured with LRP1-deficient astrocytes.

Finally, to investigate the influence of astrocytic LRP1 on neuronal activity and synaptogenesis, we assessed the cytokine release into the co-culture supernatant of LRP1-deficient or wild-type astrocytes with hippocampal neurons. However, we did not observe a significantly altered expression in the cytokine profiles of hippocampal neurons co-cultured with LRP1-deficient astrocytes in comparison to the control condition.

In summary, our results have shown that the deletion of *Lrp1* in astrocytes did not cause a reactive phenotype as characterized by an increased number of GFAP-positive astrocytes. However, other properties were altered, for example, the proliferation rate and the increased number of GLT-1-positive astrocytes. We did not observe a causal context in our investigated parameters and the decreased neuronal activity of hippocampal neurons co-cultured with LRP1-deficient astrocytes, which might relate to the limitation of this mouse model. As already described, the recombination rate and the knockout efficiency were around 55 to 77% and 82 to 90% of the cells, respectively. The proportion of non-recombined and LRP1-deficient cells still influenced the results. For the immunocytochemical stainings, only recombined cells were quantified, whereas the candidate gene expression profile analysis was performed with cell lysates of the whole cell population including non-recombined cells, which might conceal alterations between the conditions.

Despite these limitations, we identified astrocytic LRP1 as a novel modulator of astrocyte functions in the context of the tripartite synapse.

## Data Availability Statement

The raw data supporting the conclusions of this article will be made available by the authors, without undue reservation.

## Ethics Statement

The animal study was reviewed and approved by the Landesamt fuer Umweltschutz, Naturschutz und Verbraucherschutz. Written informed consent was obtained from the owners for the participation of their animals in this study.

## Author Contributions

RR and KG performed the experiments. RR, KG, and AF wrote and revised the manuscript. AS kindly provided the GLAST:CreERT2 mouse line and revised the manuscript. All authors contributed to the article and approved the submitted version.

## Conflict of Interest

The authors declare that the research was conducted in the absence of any commercial or financial relationships that could be construed as a potential conflict of interest.

## Supplementary Material

Supplementary Figure 1 Mating scheme for the newly generated inducible conditional transgenic mouse line GLAST:CreERT2xLRP1fl/fl. For the generation of the LRP1-depleted astrocyte mouse model we mated the GLAST:CreERT2 mouse line with the LRP1fl/fl mouse line. In the F1 generation triple heterozygous animals were used for further breeding. Upon the mating of the heterozygous animals of the F1 generation, 27 different genotypes of the offspring could potentially be generated. The knockout animals were characterized by the heterozygous expression of the Cre recombinase under the GLAST promotor and double floxed alleles of the tdTomato reporter and LRP1. The control has the same genetic profile, except the LRP1 alleles were not flanked by loxP sites.

Supplementary Figure 2 Exemplary recordings of MEA analysis of hippocampal neurons co-cultured with LRP1-deficient astrocytes for 21 div. The recordings of 60 electrodes on one MEA device were visualized via a heat map **(A)**. Each electrode was depicted as a rectangle and the activity was coded in a color scheme (black-strong activity; red-active; yellow-medium activity; and blue-no activity). Additionally, all occurring spikes were recorded for each electrode over the investigated time **(B)**. Furthermore, the activity of single electrodes was magnified **(C)**. The intensity of single spikes in micro Volt (μV) was presented. The number and duration of bursts were deduced by the number and frequency of spikes as it was characterized as the organized sequence of action potentials.
