# Supplementary figures and images for: Deletion of LRP1 From Astrocytes Modifies Neuronal Network Activity in an in vitro Model of the Tripartite Synapse
Source: Front Cell Neurosci. 2021 Jan 14;14:567253. doi: 10.3389/fncel.2020.567253 (PMC7842215; doi:10.3389/fncel.2020.567253)

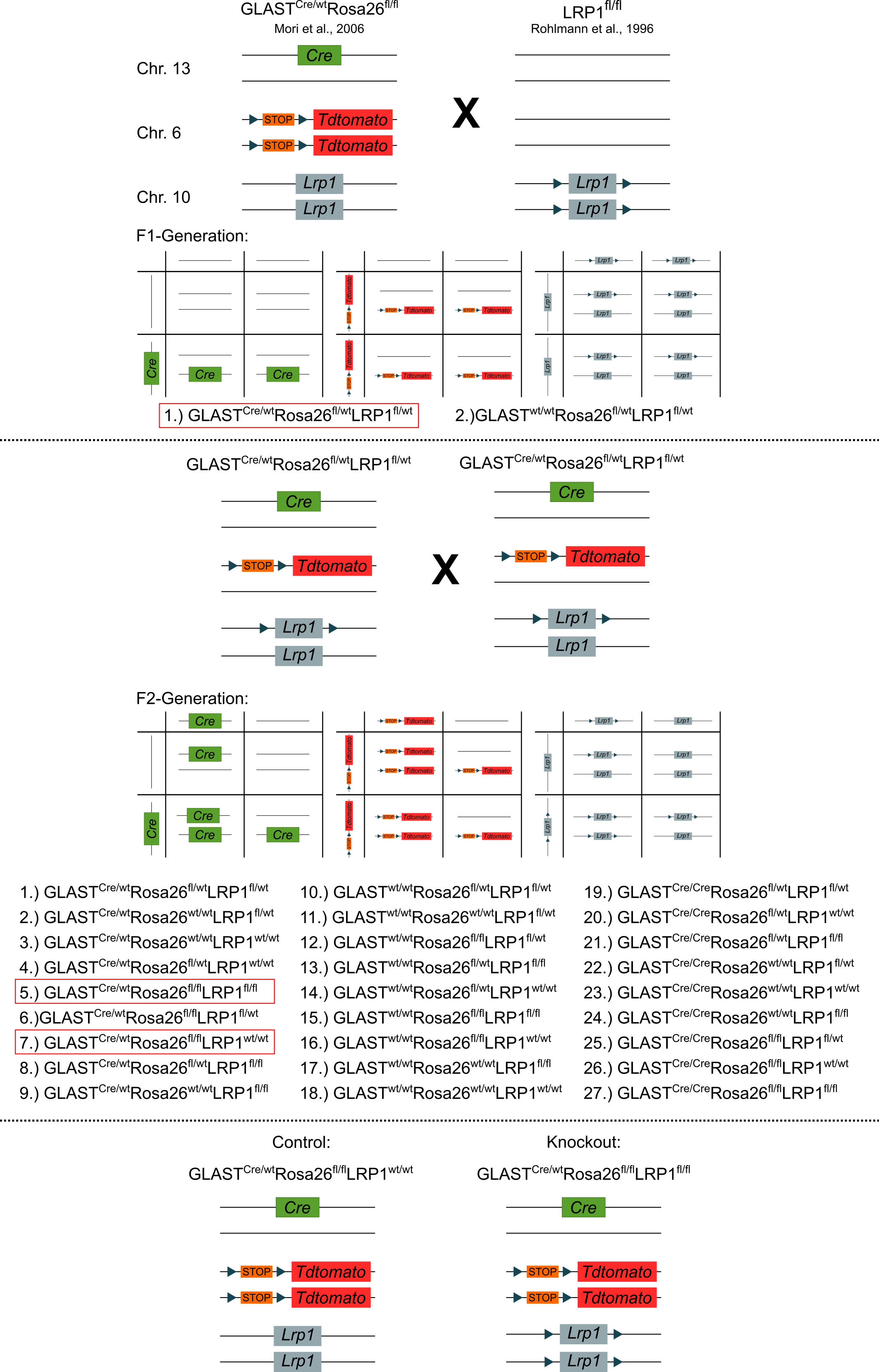

Supplement: Supplementary Figure 1 — Mating scheme for the newly generated inducible conditional transgenic mouse line GLAST:CreERT2xLRP1fl/fl. For the generation of the LRP1-depleted astrocyte mouse model we mated the GLAST:CreERT2 mouse line with the LRP1fl/fl mouse line. In the F1 generation triple heterozygous animals were used for further breeding. Upon the mating of the heterozygous animals of the F1 generation, 27 different genotypes of the offspring could potentially be generated. The knockout animals were characterized by the heterozygous expression of the Cre recombinase under the GLAST promotor and double floxed alleles of the tdTomato reporter and LRP1. The control has the same genetic profile, except the LRP1 alleles were not flanked by loxP sites. [file Image_1.TIF]

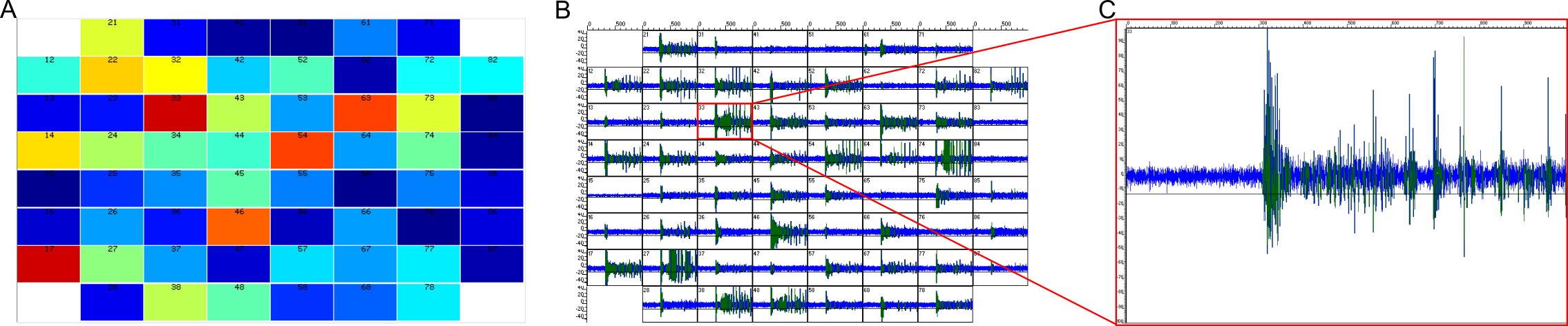

Supplement: Supplementary Figure 2 — Exemplary recordings of MEA analysis of hippocampal neurons co-cultured with LRP1-deficient astrocytes for 21 div. The recordings of 60 electrodes on one MEA device were visualized via a heat map (A). Each electrode was depicted as a rectangle and the activity was coded in a color scheme (black-strong activity; red-active; yellow-medium activity; and blue-no activity). Additionally, all occurring spikes were recorded for each electrode over the investigated time (B). Furthermore, the activity of single electrodes was magnified (C). The intensity of single spikes in micro Volt (μV) was presented. The number and duration of bursts were deduced by the number and frequency of spikes as it was characterized as the organized sequence of action potentials. [file Image_2.TIF]
